# Characterizing multi‐*PIK3CA* mutations across cancer types: Toward precision oncology

**DOI:** 10.1002/cam4.70052

**Published:** 2024-07-26

**Authors:** Kohei Nakamura, Marin Ishikawa, Ryutaro Kawano, Eriko Aimono, Takaaki Mizuno, Sachio Nohara, Shigeki Tanishima, Hideyuki Hayashi, Hiroshi Nishihara, The Keio PleSSision Group, Hiroshi Yamada, Tomoka Fujikura

**Affiliations:** ^1^ Genomics Unit, Keio Cancer Center Keio University School of Medicine Shinjuku‐ku, Tokyo Japan; ^2^ Department of Obstetrics and Gynecology Kumagaya General Hospital Kumagaya Saitama Japan; ^3^ Department of Cancer Pathology, Faculty of Medicine Hokkaido University Sapporo Hokkaido Japan; ^4^ Department of Biomedical Informatics, Communication Engineering Center, Electronic Systems Business Group Mitsubishi Electric Software Co., Ltd. Amagasaki Hyogo Japan

**Keywords:** cis, multi‐PIK3CA mutations, PI3K inhibitor, precision oncology, trans

## Abstract

**Background:**

*PIK3CA* mutations are implicated in various cancers, but the implications of multiple concurrent mutations and their orientations within the gene have not been fully explored.

**Methods:**

In this study, we analyzed multi‐*PIK3CA* mutations across a diverse pan‐cancer cohort comprising 3564 tumors.

**Results:**

Multi‐*PIK3CA* mutations were present in 10.3% of all *PIK3CA*‐mutant tumors, predominantly occurring in breast and gynecological cancers. Notably, mutations within the helical domain (E542:E545) exclusively occurred in the trans‐orientation, contrasting with mutations in the kinase ABD and C2 domains, which mainly appeared in the cis orientation.

**Conclusions:**

The distinct pattern of mutation orientations in *PIK3CA* suggests variable oncogenic potential, with helical domain mutations in the trans‐orientation potentially being less oncogenic. These findings highlight the importance of mutation orientation in the *PIK3CA* gene as potential biomarkers for targeted therapy. This understanding is crucial for designing clinical trials that leverage PI3K inhibitors, aiming for more effective and precise cancer treatment.

## Graphical abstract

This study reveals that multi‐PIK3CA mutations, found in 10.3% of PIK3CA‐mutant tumors across a broad pan‐cancer cohort, display a unique pattern of cis/trans‐orientations, with a notable distinction in the helical domain where mutations occur predominantly in trans. This suggests a lower oncogenic potential and highlights the importance of mutation location and allelic orientation in targeted cancer therapy. These insights are critical for optimizing PI3K inhibitor clinical trials, offering a path toward more effective, precision‐based treatments.

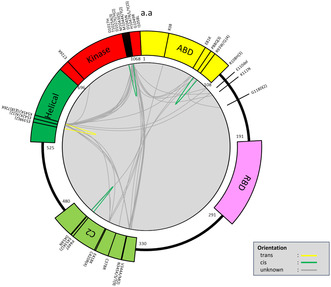

## 1 INTRODUCTION

*PIK3CA* gene is responsible for producing p110a, which is a crucial component of the PI3K lipid kinase complex. This gene is typically mutated in a wide range of cancers such as endometrial and breast cancer,^1^ leading to increased tumor growth and cancer cell signaling.^2^ Historically, PI3K has been a major therapeutic target in cancer treatment, with extensive research exploring dual PI3K/mTOR inhibitors, pan‐PI3K inhibitors, and p110a‐specific inhibitors in various types of cancers with both wild‐type and mutant *PIK3CA*.^3^, ^4^ Despite some progress in extending progression‐free survival in patients with *PIK3CA*‐mutant tumors, the development of targeted drugs has been limited owing to severe side effects, such as hyperglycemia.^5^, ^6^ Therefore, recent research has shifted focus to identifying genomic markers indicative of a high likelihood of positive treatment response, particularly in patients with *PIK3CA*‐mutant tumors.

Double or multiple *PIK3CA* mutations occur in 10%–15% of *PIK3CA*‐mutant cancers,^7^, ^8^ typically on the same allele. These multiple mutations enhance PI3K pathway signaling and are associated with an increased response to PI3K inhibitors, particularly in breast cancer, highlighting the significance of these mutations in oncogenic processes.^7^, ^8^ Consequently, investigations of PI3Ka inhibitors in early stage clinical trials across various tumor types harboring multi‐*PIK3CA* mutations have gained momentum.^6^ Initial research on *multi‐PIK3CA* mutations has predominantly focused on breast cancer; however, the frequency, mutation sites, co‐occurrence with other genomic alterations, and their links to mutational signatures in other cancer types are not well understood. Moreover, the effects of specific combinations of multi‐*PIK3CA* mutations on clinical tumor responses remain unclear.

In this study, we analyzed multi‐*PIK3CA* mutant tumors across a diverse array of cancers. We hypothesized that the frequency and patterns of multi‐*PIK3CA* mutations vary significantly across different cancer types and that these variations likely correlate with distinct clinical features or prognoses. Furthermore, we sought to investigate the co‐occurrence of multi‐*PIK3CA* mutations with other genomic alterations, postulating that these combinations may influence tumor behavior and treatment responses. Given the enhanced responsiveness to PI3K inhibitors in breast cancer with multi‐*PIK3CA* mutations, we also explored the potential for similar responses in other cancer types, which could suggest broader applications for these inhibitors. This study aimed to further our understanding of the role of multi‐*PIK3CA* mutations in various cancers and will hopefully contribute to the optimization of targeted therapies. Overall, our findings present a clearer roadmap for navigating the complex landscape of *PIK3CA* mutations in the field of oncology.

## 2 MATERIALS AND METHODS

### 2.1 Sample collection and study design

We retrospectively recruited 3564 patients with solid tumors from the Keio PleSSision Group Database (Keio University Hospital, Tokyo, Japan); all of these patients underwent a 324‐gene somatic genomic profiling test (FoundationOne® CDx) between August 2020 and September 2023. The study design was approved by the Ethics Committee of Keio University Hospital (approval number: 20211159). The requirement for informed consent was waived owing to the retrospective nature of this study. The study procedures involving human participants adhered to the principles outlined in the World Medical Association Declaration of Helsinki.

### 2.2 NGS analysis

Formalin‐fixed, paraffin‐embedded tissue samples were collected and analyzed using the FoundationOne® CDx (Foundation Medicine Inc., Cambridge, MA, USA).^9^ The types of mutations identified were short variants (base substitutions and indels), copy‐number alterations, and rearrangement events. Tumor mutational burden (TMB) was calculated as the number of somatic base substitutions or indels per megabase (Mb) of the coding region target territory and was determined to be 0.8–1.2 Mb. Samples with TMB of at least 10 mutations/Mb were classified as TMB‐high, whereas the remaining samples were classified as TMB‐low, as per the criteria set by FoundationOne® CDx. Microsatellite instability status was determined by analyzing homopolymer repeat loci. Multi‐*PIK3CA* specimens were defined as those with at least two known and likely pathogenic *PIK3CA* mutations.

### 2.3 Estimation of cis/trans‐orientation of multi‐ *PIK3CA* mutations

*PIK3CA* mutation pairs within 49 nucleotides of each other were assessed for their cis/trans‐orientation. To mitigate the confounding factors arising from potential sequencing errors, cis/trans‐calling was performed for variant pairs with over 10 read pairs supporting either the mutant or wildtype sequence at both variants. Cis support was defined as a mutation pair with reads supporting both mutant sequences. Trans‐support was defined as a mutation with read support for one mutant and one wild‐type sequence. Mutation pairs were considered cis‐oriented when more than four read pairs supported the cis status. According to the algorithm used by Foundation Medicine, Inc., mutations located more than 50 nucleotides apart could not be reliably assessed for cis/trans‐orientation; therefore, our evaluation was limited to cases within the 49‐nucleotide range.

### 2.4 Statistical analysis

Chi‐square tests were conducted to assess the significance of the observed differences in mutation frequencies between the groups. When comparisons were made between small sample sizes, Fisher's exact test was employed to determine the significance of the observed differences. Data were stratified as necessary to account for variations across different categories. In cases where the expected frequencies in any category were too low for a valid chi‐square test, a statistical comparison was not performed. A *p*‐value <0.05 was considered significant.

## 3 RESULTS

### 3.1 Pan‐cancer prevalence of *PIK3CA* mutations

**FIGURE 1:**
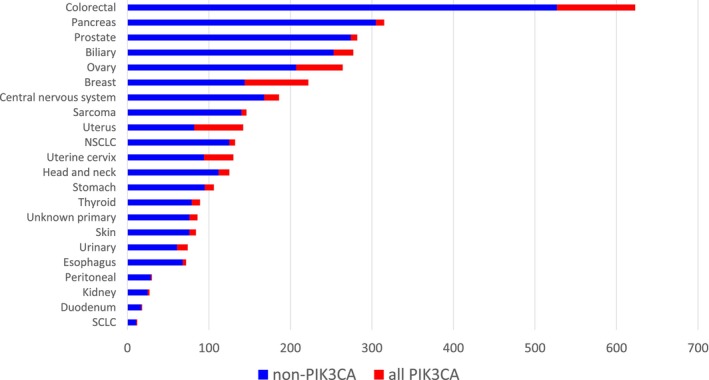
Prevalence of *PIK3CA* mutations across cancer types. *PIK3CA* mutations identified from a pan‐tumor cohort of 3564 samples were assessed for genomic patterns and rates of occurrence. Stacked bar plot shows the percentage of samples exhibiting any *PIK3CA* mutation: *PIK3CA* mutation present (teal) or absent (yellow).

**FIGURE 2:**
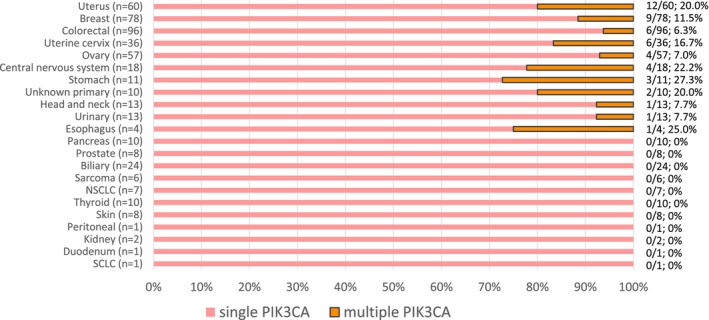
Prevalence of multi‐*PIK3CA* mutations across tumor types. Multi‐*PIK3CA* mutations identified from a pan‐tumor cohort of 474 samples with any *PIK3CA* mutations were assessed for genomic patterns and rates of occurrence. Stacked bar plot shows the percentage of samples exhibiting *PIK3CA* mutations: Single *PIK3CA* mutation (orange) and multiple *PIK3CA* mutations (blue).

Figure 1 shows the *PIK3CA* mutation status and number of patients with each cancer type. *PIK3CA* mutations were identified in 13.0% (474/3564) of samples in the pan‐cancer cohort, with the highest prevalence observed in uterine/endometrial (42.3%), breast (35.1%), cervical (27.7%) and ovarian (21.6%) cancers (Figure 1, Table S1). Multi‐*PIK3CA* mutations (two or more within one sample) were rare, identified in 1.37% of the total cohort and in 10.3% (49/474) of the samples with any PIK3CA alteration with the highest prevalence observed in uterus/endometrial (*n* = 12, 20.0%), breast (*n* = 9, 11.5%), cervical (*n* = 6, 16.7%), colorectal (*n* = 6, 6.3%), ovarian (*n* = 4, 7.0%), CNS (*n* = 4, 22.2%), stomach (*n* = 3, 27.3%), unknown primary (*n* = 2, 20.0%), urinary (*n* = 1, 7.7%), esophageal (*n* = 1, 25.0%), and head and neck (*n* = 1, 7.7%) tumors (Figure 2, Table S1). Of these, 24.5% (12/49) was TMB‐high, multi‐*PIK3CA* mutant tumors (≥10 mutations/Mb) (uterine: *N* = 6, breast: *N* = 4, uterine cervix: *N* = 1, stomach: *N* = 1).

**FIGURE 3:**
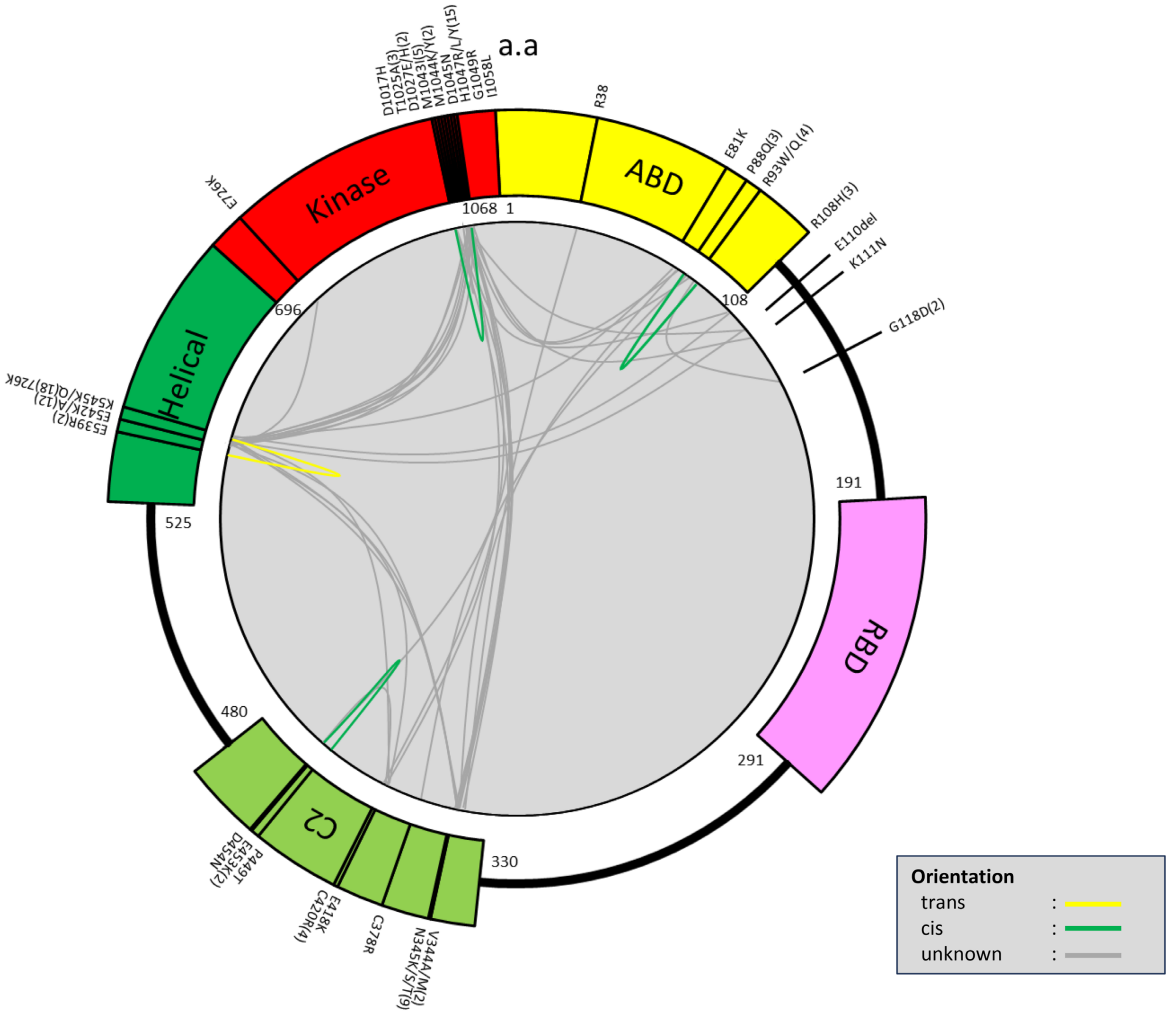
Circos plot illustrating the spatial relationship between mutation sites and domains within the *PIK3CA* gene. Each arc represents a specific mutation site, and its position along the circular plot corresponds to its genomic location within the gene. The colors and connecting lines indicate the presence of mutations in different *PIK3CA* domains, providing a visual representation of the distribution and associations of mutations in the gene.

We also investigated possible co‐mutation patterns. In most double‐*PIK3CA*‐mutant tumors (45/49), one of the mutations was either a helical or kinase domain‐major hotspot mutation (involving E542, E545, or H1047) (Table S2), which are the most common alterations in single‐mutant tumors. We observed various mutational combinations within *PIK3CA* mutation domains. Specifically, we identified 10 cases with helical‐kinase mutations, 8 cases with C2‐kinase mutations, 7 cases with helical‐helical mutations, 4 cases with ABD‐kinase mutations, 4 cases with C2‐helical mutations, 3 cases with kinase‐kinase mutations, 2 cases with ABD‐helical mutations, 2 cases with other‐kinase mutations, 2 cases with C2‐C2 mutations, 2 cases with ABD‐C2 mutations, 1 case with other‐helical mutations, 1 case with ABD‐C2‐kinase mutations, 1 case with ABD‐C2‐helical‐kinase mutations, and 1 case with C2‐helical‐kinase mutations (Table 1). The relationship between these mutation sites and domains is depicted in Figure 3.

**TABLE 1:** Relationship of multiple mutations within PIK3CA domains in cases with multiple PIK3CA mutations.

ABD: actin‐binding domain	
Domain pairs	*N*
Helical‐kinase	10
C2‐kinase	8
Helical‐helical	7
ABD‐kinase	4
C2‐helical	4
Kinase‐kinase	3
ABD‐helical	2
Other‐kinase	2
C2‐C2	2
ABD‐C2	2
Other‐helical	1
ABD‐C2‐kinase	1
ABD‐C2‐helical‐kinase	1
C2‐helical‐kinase	1
ABD‐other‐helical	1

### 3.2 Clonal and subclonal patterns in multi‐ *PIK3CA* mutations

**FIGURE 4:**
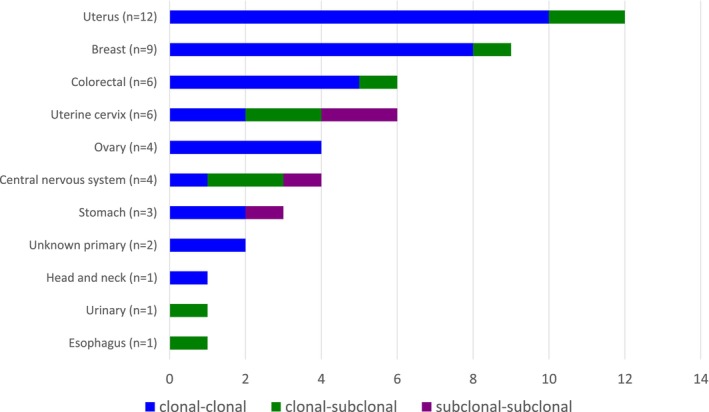
Distribution of clonal and subclonal mutation patterns across various cancer types. In the bar chart, blue bars represent cases with clonal‐clonal mutation patterns, orange bars represent cases with clonal‐subclonal mutation patterns, and gray bars represent cases with subclonal‐subclonal mutation patterns.

In our pan‐cancer cohort of 49 patients with multiple *PIK3CA* mutations, 88% (43 patients) exhibited double‐*PIK3CA* mutations. Of these, five cases had three mutations, and one case had five mutations. We conducted computational analysis to evaluate the clonal status of these mutations. In accordance with the Foundation of Medicine criteria, mutations were defined as “subclonal” if the variant allele frequency was less than one‐tenth of the estimated tumor purity. Among the 43 double‐*PIK3CA* cases, 77% (33/43) had clonal (clonal‐clonal pairs) mutations, while 14% (6/43) had a combination of one clonal and one subclonal mutation (clonal‐subclonal pairs); however, the remaining 9% (4/43) had both subclonal mutations. Among the five cases with three *PIK3CA* mutations, two had all‐clonal mutations, two had two clonal and one subclonal mutations, and one had one clonal and two subclonal mutations. A total of five *PIK3CA* mutation clones were identified, including one clonal and four subclonal mutations. The distribution of clonal and subclonal patterns across different cancer types is shown in Figure 4.

### 3.3 Orientation of multiple *PIK3CA* mutations

Allelic configuration analysis of mutations, particularly in formalin‐fixed, paraffin‐embedded samples, poses significant challenges^7^ when mutations are located beyond the span of a single sequencing read. Considering these challenges, we focused on a subset of 11 cases with multi‐*PIK3CA*, where each pair of mutations was situated within 49 nucleotides of each other. For the double mutations p.E542 and E545, all six cases exhibited a trans‐configuration; however, a cis configuration was observed in the remaining four cases (Table 2). In the case of three *PIK3CA* mutations (E542K, E545Q, and E545K), the E542K mutation is in trans‐orientation with respect to both E545Q and E545K mutations. Notably, the E545Q and E545K mutations cannot physically occur in cis orientation on the same allele because of simultaneous substitutions at the same residue. This description aims to convey the complexity of mutational events in *PIK3CA*. A more accurate interpretation suggests that these represent complex mutational events rather than independent mutations in close proximity. Further analysis revealed domain‐specific orientations of these multiple mutations within different protein domains. For instance, D1029 and D1045 mutations in the kinase domain, R88 and R93 mutations in the ABD domain, and E453 and D454 in the C2 domain were in cis orientation. In contrast, mutations within the helical domain, such as E542 and E545, were in trans‐orientation. Figure 3 presents this domain‐specific pattern of mutation orientations, providing a comprehensive visual overview of these allelic configurations across the dataset. Additionally, Figure 3 presents the relationship between the cis‐ and trans‐configurations for each of the 49 mutations.

**TABLE 2:** Prediction of cis/trans‐orientation for double *PIK3CA*‐mutated tumors.

Mutation pairs	Total	Cis	Trans	Cis (%)
E542K:E545K	6	0	6	0.00
R88Q:R93Q	1	1	0	100.00
E453K:D454N	1	1	0	100.00
M1043I:N1044Y	1	1	0	100.00
D1017H:D1029H	1	1	0	100.00
All	10	4	6	40.00

### 3.4 PIK3CA mutations and co‐occurrence with other genomic alterations

To examine the mutational landscape of tumors with multi‐*PIK3CA* mutations, we performed a co‐occurrence analysis of alterations in other genes associated with the PI3K/AKT pathway, including *PTEN*, *PIK3R1*, *AKT1*, *AKT2*, *AKT3*, and *MTOR*. This analysis revealed that while *PTEN* mutations occurred in both single and multiple *PIK3CA* mutation cohorts, their distribution varied across different cancer types. Notably, in colon, ovarian, cervical, and breast cancers, patients with multiple *PIK3CA* mutations exhibited a distinct pattern of *PTEN* mutation co‐occurrence compared with those with a single *PIK3CA* mutation. Specifically, among patients with colon cancer, only 6 out of 617 cases (0.96%) with multiple *PIK3CA* mutations exhibited *PTEN* mutations compared with 6 out of 90 cases (6.67%) with a single *PIK3CA* mutation. In ovarian cancer, 4 out of 260 cases (1.52%) with multiple *PIK3CA* mutations showed *PTEN* mutations compared with 4 out of 53 cases (7.55%) with a single mutation. In cervical cancer, 6 out of 124 cases (4.62%) with multiple *PIK3CA* mutations exhibited *PTEN* mutations compared with 3 out of 42 cases (7.14%) with a single mutation. Finally, in breast cancer, 9 out of 213 cases (4.05%) with multiple *PIK3CA* mutations had *PTEN* mutations compared with 7 out of 70 cases (10.00%) with a single mutation. These findings suggest that the presence of multiple *PIK3CA* mutations correlates with a lower co‐occurrence rate of *PTEN* mutations in these specific cancer types.

During the comprehensive analysis including all cancer types, the chi‐square test did not demonstrate a significant difference in the prevalence of *PTEN* mutations between patients with a single *PIK3CA* mutation (36 of 425 cases) and those with multiple *PIK3CA* mutations (6 of 49 cases). Nonetheless, distinct trends were observed when the data were stratified according to specific cancer type. In addition, we examined the co‐occurrence of other key driver gene mutations in tumors, specifically focusing on genes with a mutation prevalence of at least 30% in cases where genomic alterations were reliably identified. This analysis included *KRAS* mutations, which were detected in 9 of 47 cases with multiple *PIK3CA* mutations and in 97 of 290 cases with a single *PIK3CA* mutation within the same organ group where multiple *PIK3CA* mutations were observed. Fisher's exact test yielded an odds ratio of approximately 0.45 and a *p*‐value of approximately 0.045. These results initially suggested that the co‐occurrence rate of *KRAS* mutations might be lower in cases with multiple *PIK3CA* mutations than in those with a single *PIK3CA* mutation. However, when adjusting for cancer type as a covariate in a multivariate analysis, no significant difference was observed in the co‐occurrence rate of *KRAS* mutations between patients with multiple and single *PIK3CA* mutations. This adjustment reflects the diverse impact of cancer type on mutation co‐occurrence and highlights the complexity of interpreting genomic interactions in cancer. No significant differences were observed in the co‐occurrence with other oncogenes such as *BRAF* and *ERBB2*.

## 4 DISCUSSION

In this study, we conducted a comprehensive analysis of *PIK3CA* mutations across various cancer types, with a focus on cases exhibiting multiple *PIK3CA* mutations. Our findings highlighted several crucial aspects of *PIK3CA* mutations, factors affecting mutational distributions in multiple cancers, and their potential implications in cancer biology. Our analysis revealed a pan‐cancer prevalence of *PIK3CA* mutations in 13.0% of the samples, with the highest occurrence observed in uterine/endometrial, breast, cervical, and ovarian tumors. These findings align with those of a previous study emphasizing the role of *PIK3CA* mutations in diverse cancer types.^10^ Notably, we identified a subset of patients with multiple *PIK3CA* mutations, albeit at a low frequency (1.37% of the total cohort). This subset warrants further exploration as it may possess distinctive clinical and molecular characteristics that could enhance our understanding of *PIK3CA* and its possible role as a therapeutic target.

A pivotal aspect of our study was the assessment of clonal and subclonal patterns in patients harboring multiple *PIK3CA* mutations. Our analysis, in alignment with the Foundation Medicine's criteria, revealed that the majority of double‐*PIK3CA* mutation cases had both mutations classified as clonal, implying a potential driving role of these alterations in tumorigenesis. Nevertheless, a subset of cases exhibited a combination of clonal and subclonal mutations, highlighting the intricacy of *PIK3CA* mutation patterns within individual tumors and cancer types. These findings emphasize the importance of accounting for clonal heterogeneity while evaluating the functional significance of *PIK3CA* mutations.

Another critical aspect of our analysis involved elucidating the cis/trans‐orientation of multi‐*PIK3CA* mutations. Our approach was restricted to mutations located within 49 nucleotides of each other, limiting our in‐depth assessment to 11 of 49 cases with multiple mutations. Although this provided valuable insights into the allelic configuration of these mutations, it also highlighted a considerable constraint. Future studies may benefit from the application of long‐read sequencing technologies, which may enable a more comprehensive evaluation of the mutational landscape in the *PIK3CA* gene.

Studies have reported that the overall consequence of cis mutations is a phenotype of enhanced oncogenicity and increased sensitivity to PI3Kα inhibitors.^7^, ^11^ Although our sample size was limited, the observation that all cases with the E542:E545 mutation were in the trans‐orientation suggests that the E542:E545 combination tends to occur in trans‐orientations, potentially conferring a low level of oncogenicity. However, further validation using a large sample size is essential to confirm this observation and its functional implications. Notably, as can be seen in Figure 3, multiple *PIK3CA* mutations within the same domain exhibited distinct orientations based on the domain type. For example, mutations within the kinase ABD and C2 domains were all in cis orientation, whereas those within the helical domain were in trans‐orientation. This suggests that in cases of multiple PIK3CA mutations, the cis/trans‐orientation tends to vary based on the domain, thereby indicating a potential domain‐specific pattern of mutation orientation.

Experimental models of the oncogenic potential of double‐*PIK3CA* mutations^7^, ^8^ have shown that these mutations rarely co‐occur with other major oncogenes and tumor suppressors, suggesting a distinct pathway for tumorigenesis; thus, we propose a continuum model in which varying oncogene dosages impact cancer development differently.^12^ Understanding specific combinations of *PIK3CA* mutations and their functional consequences is crucial for elucidating the complex interplay between genomic alterations within tumors.

We also investigated the co‐occurrence of *PIK3CA* mutations with alterations in other genes involved in the PI3K/AKT pathway. We did not observe a significant difference in the prevalence of *PTEN* mutations between cases with a single *PIK3CA* mutation and those with multiple *PIK3CA* mutations across all cancer types, likely because *PTEN* mutations are common driver mutations in endometrial cancer; therefore, their occurrence is more likely to be tumor‐specific rather than being associated with the number of *PIK3CA* mutations. Furthermore, no significant differences were observed in other PI3K/AKT pathway‐related genes, which may be due to the limited number of cases, suggesting that a large cohort is necessary for a conclusive analysis. These results suggest that, while the observed trends indicate potential differences in the prevalence of *PTEN* mutations between single and multiple *PIK3CA* mutation cases, the absence of *PTEN* mutations in multiple mutation groups in some cancer types and small sample sizes in groups likely influenced the ability to achieve significance of the results. Therefore, further research with large cohorts is essential to robustly evaluate the association between the *PIK3CA* single/multiple mutation status and the prevalence of *PTEN* mutations across various cancer types. However, further stratification of the data by cancer type indicated potential trends for specific cancer types. Notably, patients with endometrial cancer exhibited a high proportion of *PTEN* mutations when they had multi‐*PIK3CA* mutations as opposed to only a single mutation, although the difference was not significant. These observations highlight the need for large cohorts and tumor‐specific considerations while evaluating the association between the PIK3CA mutation status and *PTEN* alterations.

Although our study presents some interesting findings, it was limited by the relatively small sample size of cases with multiple *PIK3CA* mutations in some cancer types. Additionally, owing to the nature of this multi‐institutional study, which primarily focused on genomic analysis, we were unable to incorporate functional biology experiments or detailed clinical data such as treatment outcomes or prognosis. Moreover, the functional implications of clonal/subclonal patterns and cis/trans‐orientations of *PIK3CA* mutations, inferred from previous literature, were not directly validated through in vitro or in vivo experiments in our study. This represents a significant limitation, as functional experiments are essential for understanding the distinct roles these genomic configurations may play in tumorigenesis and oncogenicity. Furthermore, the lack of access to comprehensive clinical data warrants future studies to explore the clinical significance of multi‐*PIK3CA* mutations and their patterns, potentially in conjunction with treatment response and patient outcomes. Therefore, further research is warranted to explore the potential clinical implications and therapeutic responses associated with specific mutation orientations (cis/trans) in *PIK3CA*.

In conclusion, our study provides a comprehensive analysis of *PIK3CA* mutations in various cancer types, shedding light on the clonal and subclonal patterns, allelic orientations, and the co‐occurrence of PI3K/AKT pathway alterations. These findings significantly contribute to the understanding of multi‐*PIK3CA* mutations in cancer and their potential as biomarkers for targeted drug therapies. As sequencing technologies evolve further and our understanding of cancer biology deepens, conducting further research studies on large cohorts and using long reads will refine our knowledge and contribute to future oncological advancements.

## AUTHOR CONTRIBUTIONS

**Kohei Nakamura:** Conceptualization (lead); writing – original draft (lead). **Marin Ishikawa:** Data curation (equal); resources (equal). **Ryutaro Kawano:** Data curation (equal); resources (equal). **Eriko Aimono:** Data curation (equal); resources (equal). **Takaaki Mizuno:** Data curation (equal); resources (equal). **Sachio Nohara:** Data curation (equal); resources (equal). **Shigeki Tanishima:** Data curation (equal); resources (equal). **Hideyuki Hayashi:** Data curation (equal); resources (equal). **Hiroshi Nishihara:** Conceptualization (equal); writing – review and editing (equal).

## FUNDING INFORMATION

This study was supported by the Japan Agency for Medical Research and Development (AMED) (Grant Number JP23ck0106872), JSPS KAKENHI (Grant‐in‐Aid for Scientific Research(C)) (Grant number 23K08829), Takeda Science Foundation (Medical Research Grants), Cell Science Research Foundation, Kanzawa Medical Research Foundation, and Uehara Memorial Foundation.

## CONFLICT OF INTEREST STATEMENT

Sachio Nohara and Shigeki Tanishima are employees of the Mitsubishi Electric Software Corporation. Sawa Nohara (wife of Sachio Nohara) is an employee of the Kaina Home Care Station. Naomi Tanishima (wife of Shigeki Tanishima) is an employee of Inoue Co., Ltd. Hideaki Tanishima (Shigeki Tanishima's son) is an employee of Marui & Co., Ltd. Mai Tanishima is an employee of Konan Medical Center. Takaaki Mizuno is an employee of Rakuten Medical K.K. Takaaki Mizuno is from Clinial Co., Ltd (corporate stocks). The other authors declare no conflicts of interest.

## ETHICS STATEMENT

Approval of the Research Protocol by an Institutional Review Board: This study was part of a research project approved by the Ethics Committee of Keio University Hospital (approval number: 20211159). The procedures involving human participants complied with the principles of the Declaration of Helsinki.

## CONSENT

Informed consent was obtained from all patients.

## Supporting information


**Table S1:** Prevalence of single and multi‐*PIK3CA* mutated tumors across different tumor types.

**Table S2:** Prevalence of specific pairs of *PIK3CA* mutation types across different tumor types.

## Data Availability

The data that support the findings of this study are not available, as the study participants did not consent to the public sharing of their data.
